# Supplementary material for: The genome sequence of the Small Fan-footed Wave moth, Idaea biselata (Hufnagel, 1767)
Source: Wellcome Open Res. 2025 Feb 19;10:78. [Version 1] doi: 10.12688/wellcomeopenres.23706.1 (PMC12853022; doi:10.12688/wellcomeopenres.23706.1)
Supplement: Peer review history

# Reviewer report: Reviewer response for version 1

**DOI:** 10.21956/wellcomeopenres.26154.r140286

**Published:** 2026-01-30

**Authors:** Shangzhe Zhang

**Affiliations:** 1 University of St Andrews Library, St Andrews, Scotland, UK

The data note presented a high-quality genome assembly of the Small Fan-footed Wave moth, as part of the ToL project. The methods are robust and the assembly metrics are well-presented. I only have a few concerns regarding the clarity of this manuscript.

 I noticed that the authors mentioned that the assembly has 380 scaffolds but all diagrams showed 381 scaffolds, is it because that the mitochondrial scaffold was not removed in these diagrams?

 "While not fully phased, the assembly deposited is of one haplotype. Contigs corresponding to the second haplotype have also been deposited." Here more clarifications are needed -- the authors mentioned that the primary contig assembly was selected and used for scaffolding -- which means no phasing was performed in assembling.

Are sufficient details of methods and materials provided to allow replication by others?

Yes

Is the rationale for creating the dataset(s) clearly described?

Yes

Are the datasets clearly presented in a useable and accessible format?

Yes

Are the protocols appropriate and is the work technically sound?

Yes

Reviewer Expertise:

Genomics, Evolutionary biology

I confirm that I have read this submission and believe that I have an appropriate level of expertise to confirm that it is of an acceptable scientific standard.

# Reviewer report: Reviewer response for version 1

**DOI:** 10.21956/wellcomeopenres.26154.r145303

**Published:** 2026-01-28

**Authors:** Axel Künstner

**Affiliations:** 1 University of Lübeck, Lübeck, Germany

This genome assembly paper for *Idaea biselata* (Small Fan-footed Wave moth) presents a high-quality chromosomal-level reference genome as part of the Darwin Tree of Life project.

**Strengths:**

- **Excellent assembly quality**: Scaffold N50 of 31 Mb, QV of 57.4, and 97.24% scaffolded into 13 chromosomes meets rigorous EBP standards (5.C.Q57)
- **Comprehensive methodology**: Clear documentation of PacBio HiFi, Hi-C scaffolding, and manual curation with well-described protocols
- **Species-specific annotation**: RNA-seq data from the same specimen improves gene model accuracy (11,310 protein-coding genes)
- **Complete mitochondrial genome**: 17.33 kb mitogenome adds value for phylogenetic studies
- **Strong BUSCO scores**: 94.6% completeness validates assembly quality

**Limitations:**

- **Limited biological context**: As reviewer Dey notes, the paper would benefit from discussing how this genome advances geometrid moth biology, adaptation, or phylogenomics
- **Minor inconsistencies**: Reviewer Huang identifies scaffold count discrepancies (380 vs 381) between text and figures
- **Missing validation details**:
  - RNA-seq mapping rates to validate gene models not provided
  - Mitogenome circularity not explicitly confirmed
  - Software table incomplete (JBrowse2, Ensembl Genebuild missing)

**Overall Assessment:**

 This is a technically sound resource paper with robust genomic data that fills an important gap for Geometridae genomes. The assembly quality is excellent and methodology reproducible. However, it reads as purely descriptive without contextualizing the genome's utility for addressing specific biological questions about this species or geometrid moths generally. For a data note, this is acceptable, but adding even 1-2 paragraphs on potential applications would significantly enhance impact.

Are sufficient details of methods and materials provided to allow replication by others?

Yes

Is the rationale for creating the dataset(s) clearly described?

Yes

Are the datasets clearly presented in a useable and accessible format?

Yes

Are the protocols appropriate and is the work technically sound?

Yes

Reviewer Expertise:

Cancer genomics, Microbiome

I confirm that I have read this submission and believe that I have an appropriate level of expertise to confirm that it is of an acceptable scientific standard.

# Reviewer report: Reviewer response for version 1

**DOI:** 10.21956/wellcomeopenres.26154.r139606

**Published:** 2026-01-21

**Authors:** Yu-Feng Huang

**Affiliations:** 1 National Chung Hsing University, Taichung City, Taiwan; 2 Computer Science and Engineering, Yuan Ze University (Ringgold ID: 34895), Zhongli District, Taoyuan City, Taiwan

This Data Note presents a high-quality reference genome assembly for the Small Fan-footed Wave moth, *Idaea biselata*, generated using PacBio HiFi and Illumina Hi-C data. The assembly metrics are robust, with a scaffold N50 of 31.0 Mb and a consensus quality (QV) of 57.4. The authors have successfully assigned 97.24% of the sequence to 13 chromosomal pseudomolecules, representing the 12 autosomes and the Z sex chromosome. Notably, this study incorporates species-specific RNA-Seq data to support genome annotation, identifying 11,310 protein-coding genes.

 While the data quality is high and meets Earth BioGenome Project standards, there are distinct inconsistencies regarding scaffold counts between the text and figures. Additionally, minor clarifications regarding mitogenome circularization, annotation statistics, and software documentation are required to ensure the manuscript's precision.

**Major Comments**

**1. Discrepancy in Scaffold Counts** There is a clear numerical inconsistency regarding the total number of scaffolds in the assembly. The main text, and Table 2 explicitly state that the final assembly consists of **380** sequence scaffolds. However, the legend for Figure 2 , the header for Figure 3 , and the legend for Figure 4 all list the total scaffold count as **381**. Is it possible that the inclusion of mitochondrial genome?

**Minor Comments**

**2. Gene Annotation Validation & Metrics** This study commendably includes species-specific RNA-Seq data (ERR12245555) derived from abdomen tissue. The annotation methods state this transcriptomic data was used for alignment. While the methodology is sounder than protein-homology alone, the manuscript lacks specific metrics on the utility of this RNA-Seq data. To validate the annotation quality, please briefly include the mapping rate of the RNA-Seq reads to the final assembly. High mapping rates would empirically confirm the structural accuracy of the gene models generated.

**3. Mitogenome Circularization Verification** The mitochondrial genome (17.33 kb) was assembled using MitoHiFi, which the text states ensures "general quality". As noted in reviews of similar manuscripts, the text does not explicitly confirm that the assembly was verified as circular. Please confirm if the circular topology was validated (e.g., by checking for overlapping ends) and if the sequence was rotated to a standard start feature (e.g., *tRNA-Phe* or *COX1*) to ensure it represents the complete organelle genome.

**4. Software Tools (Table 4)** Table 4 lists the software versions used. There are minor omissions compared to the methods section. **JBrowse2**, mentioned in the curation section , and the **Ensembl Genebuild** system, mentioned in the annotation section, are not listed in the software table. Please audit Table 4 and add these missing tools with their respective versions/sources.

Are sufficient details of methods and materials provided to allow replication by others?

Yes

Is the rationale for creating the dataset(s) clearly described?

Yes

Are the datasets clearly presented in a useable and accessible format?

Yes

Are the protocols appropriate and is the work technically sound?

Partly

Reviewer Expertise:

Bioinformatics, Cancer Bioinformatics, Mitogenomics, Genome sequencing, assembly and gene annotation.

I confirm that I have read this submission and believe that I have an appropriate level of expertise to confirm that it is of an acceptable scientific standard, however I have significant reservations, as outlined above.

# Reviewer report: Reviewer response for version 1

**DOI:** 10.21956/wellcomeopenres.26154.r140289

**Published:** 2026-01-10

**Authors:** Pritha Dey

**Affiliations:** 1 National Centre for Biological Sciences-TIFR, Bengaluru, India

This manuscript presents a chromosomal-scale genome assembly of Idaea biselata (Lepidoptera: Geometridae), generated from a single male individual. The methods are clear and the inclusion of mitochondrial genome further enhances the utility of the dataset

 The availability of a reference genome for I. biselata is a valuable contribution, particularly given the underrepresentation of Geometridae in genomic resources, and will be useful for studies in lepidopteran evolution, ecology, and comparative genomics.

 However, the manuscript could be strengthened by placing the genome in a broader biological context. A short discussion on how this genome resource may advance studies on geometrid moth biology, adaptation, or phylogenomics would enhance its impact.

Are sufficient details of methods and materials provided to allow replication by others?

Yes

Is the rationale for creating the dataset(s) clearly described?

Yes

Are the datasets clearly presented in a useable and accessible format?

Yes

Are the protocols appropriate and is the work technically sound?

Yes

Reviewer Expertise:

NA

I confirm that I have read this submission and believe that I have an appropriate level of expertise to confirm that it is of an acceptable scientific standard.
